# Metagenomic Next-Generation Sequencing Diagnosis of Streptococcus agalactiae Meningitis in a Diabetic Patient

**DOI:** 10.7759/cureus.94759

**Published:** 2025-10-16

**Authors:** Xiaoyan He, Mao Wang, Ting Sun, Jiaoqi Tang, Yiqian Zeng

**Affiliations:** 1 Emergency Department, Zhuzhou Central Hospital, Zhuzhou, CHN

**Keywords:** immunocompromised, meningitis, metagenomic next-generation sequencing (mngs), myelitis, streptococcus agalactiae

## Abstract

*Streptococcus agalactiae *meningitis is primarily observed among neonates and is uncommon in adults. We present a rare case of *Streptococcus agalactiae *meningitis in an adult. The patient was a 74-year-old male with a history of gastric perforation surgery, chemotherapy for neck lymphoma, and hypertension. He presented to the emergency department with an 11-day history of neck pain, one day of limited mouth opening and dysphagia, and eight hours of altered consciousness. On examination, he exhibited impaired consciousness (Glasgow Coma Scale score: 8) and cervical rigidity. He was intubated and received oxygen therapy. Laboratory findings revealed elevated infection markers and turbid cerebrospinal fluid (CSF). Metagenomic next-generation sequencing (mNGS) of the CSF detected *Streptococcus agalactiae*. Enhanced MRI of the head and neck showed a small subdural effusion, spinal cord edema at C5-C6, thickening and enhancement of the anterior and posterior longitudinal ligaments and meninges at the skull base, suggestive of *Streptococcus agalactiae* infection. After 10 days of anti-infective treatment with ceftriaxone, the patient's condition improved, and he was transferred to a local hospital for continued management.

## Introduction

*Streptococcus agalactiae* (Group B *Streptococcus* (GBS)) is the leading pathogen responsible for meningitis and sepsis in neonates [1]. Regarding CNS infection, the incidence of GBS meningitis in adults appears to be exceptionally low [2]. Some studies suggest that patients who are immunocompromised, elderly, diabetic, or with endocarditis are at particular risk of GBS meningitis [2,3]. Conventional diagnostic methods for meningitis, such as cerebrospinal fluid (CSF) culture and Gram staining, are time-consuming and may yield false-negative results, especially in patients with prior antibiotic exposure. Metagenomic next-generation sequencing (mNGS) is an emerging, culture-independent technique that enables rapid and unbiased pathogen identification from clinical samples. It is particularly valuable for diagnosing rare, fastidious, or unexpected pathogens in complex clinical scenarios [4,5]. Here, we report a rare case of *Streptococcus agalactiae* meningitis in an elderly diabetic patient, which was rapidly and definitively diagnosed using mNGS of the CSF after initial conventional diagnostics proved inconclusive.

## Case presentation

A 74-year-old male had a history of gastric perforation surgery in 2004. In 2016, he was diagnosed with neck lymphoma and received chemotherapy, with subsequent re-examinations reported as normal; however, detailed records were unavailable. He had a 10-year history of hypertension managed with antihypertensive medication. One month prior to admission, he sustained a knife cut to his left hand at home and did not receive tetanus prophylaxis. On November 9, 2023, he presented to the emergency department with an 11-day history of neck pain, one day of trismus and dysphagia, and eight hours of altered consciousness. Vital signs on arrival were: temporal temperature 37.7°C, blood pressure 191/83 mmHg, pulse 96 beats/minute, respiratory rate 22 breaths/minute, and oxygen saturation 94% on room air. He was drowsy with a Glasgow Coma Scale (GCS) score of 8/15. Brudzinski's and Kernig's signs were positive, and limb muscle tone was increased. Initial laboratory tests revealed leukocytosis, hyponatremia, hypoalbuminemia, hypoproteinemia, and normal liver and kidney function. A pre-admission head and neck CT scan from another hospital showed no intracranial abnormalities but noted cervical osteophyte formation. Initial differential diagnoses included tetanus and intracranial infection. Management included endotracheal intubation for oxygen therapy, nasogastric feeding, administration of tetanus immunoglobulin and antitoxin, and intravenous antibiotics (ceftriaxone 2 g every 12 hours plus penicillin G 2.4 million units every eight hours). Sedative and analgesic therapy was initiated, and enoxaparin was administered subcutaneously for deep vein thrombosis prophylaxis. On November 10, 2023, a lumbar puncture was performed. Cerebrospinal fluid (CSF) was turbid with an opening pressure of 230 mmH2O. CSF analysis showed elevated white cell count, elevated protein, and decreased glucose levels. Concurrent findings included hyperglycemia (fluctuating between 9.5-16.5 mmol/L), glycated hemoglobin (HbA1c) of 7.0%, and elevated procalcitonin and interleukin-6 levels (Table 1).

**Table 1:** Laboratory workup

Serum test	2023-11-10	2023-11-20	Reference range	Unit
White blood cells	27.58	7.68	3.5-9.5	10^9^/L
Neutrophil ratio	96.3	80.5	40-75	%
Hemoglobin	111	107	130-175	g/L
Platelets	420	346	125-350	10^9^/L
Sodium	130.6	140	137-147	mmol/L
Potassium	4.67	4.6	3.5-5.3	mmol/L
Chloride	98.8	101.1	99-110	mmol/L
Calcium	1.93	2.0	2.11-2.52	mmol/L
Blood urea nitrogen	7.86	4.12	3.6-9.5	mmol/L
Creatinine	78	59	57-111	µmol/L
Aspartate transaminase	12	28	15-40	U/L
Alanine transaminase	34	66	9-50	U/L
Total bilirubin	3.3	3.5	0-26	µmol/L
Direct bilirubin	2.1	1.9	0-8	µmol /L
Total protein	56.6	58.9	65-85	g/L
Albumin	32.6	32.4	40-55	g/L
Glycated hemoglobin	7	-	3.6-6.0	%
Procalcitonin	4.74	0.1	0-0.5	ng/mL
Interleukin-6	209.3	7.6	0-7	pg/mL

**Figure 1:**
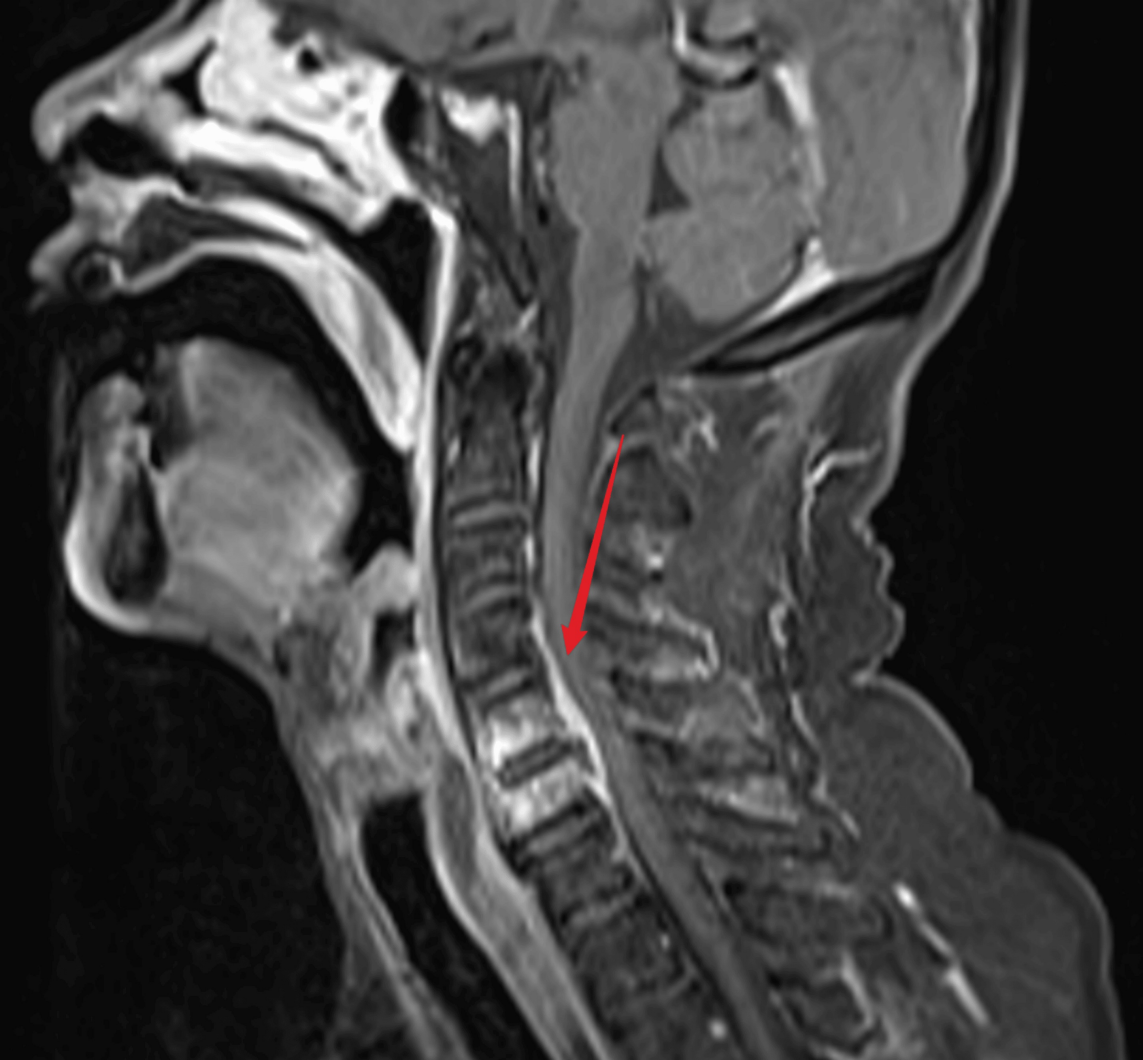
C5-C6 spinal cord edema

**Figure 2:**
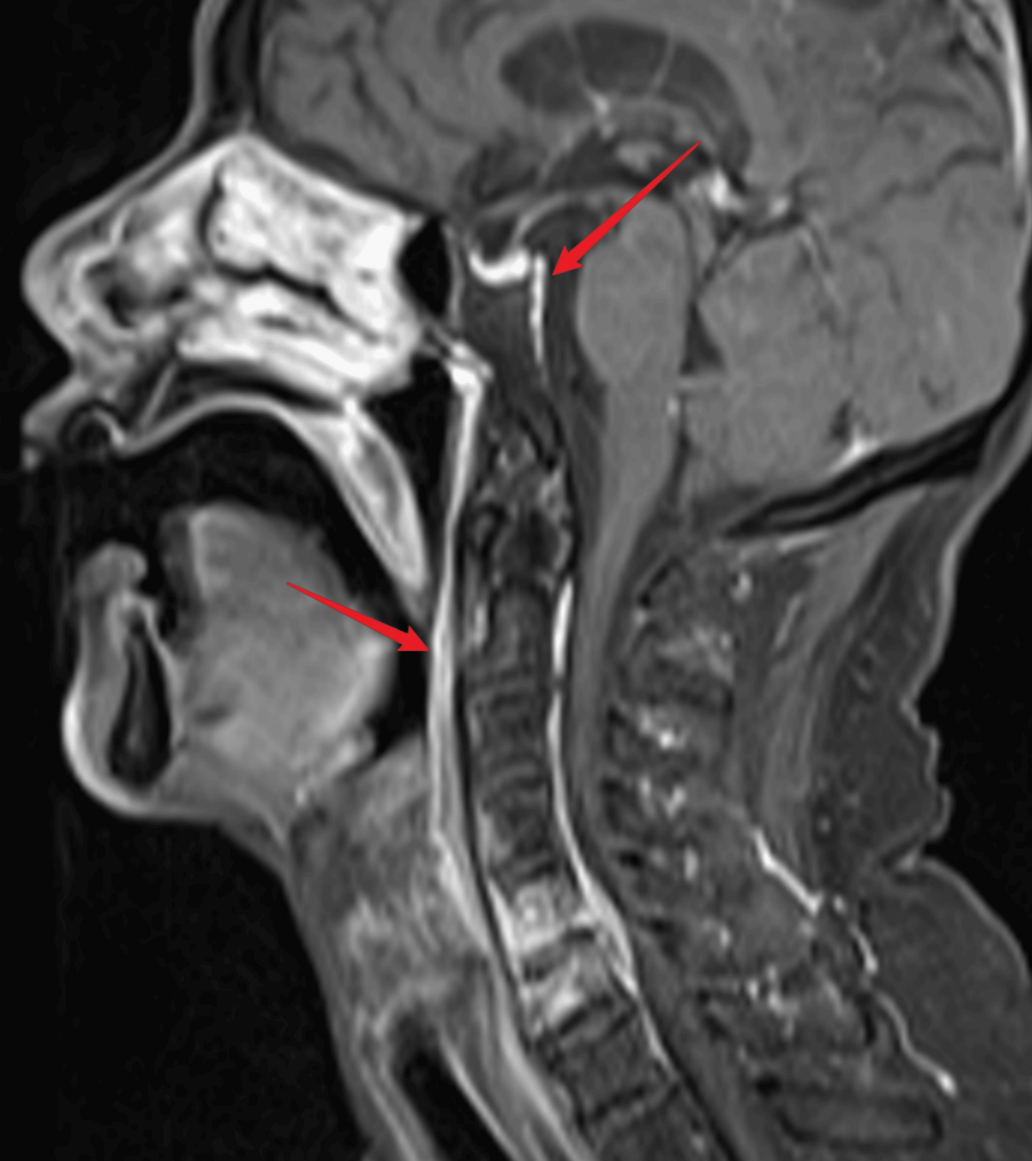
Thickening and enhancement of the anterior and posterior longitudinal ligaments and meninges at the skull base

The patient's hyponatremia is considered to be related to previous difficulty opening the mouth and reduced oral intake. After hospitalization, enteral nutrition via a nasogastric tube was administered. The serum sodium level returned to normal within two days, with a value of 141.6 mmol/L. The electrocardiogram was normal. Echocardiography revealed diastolic dysfunction but no valvular vegetations. mNGS of CSF performed on November 13, 2023, identified *Streptococcus agalactiae* with 28 sequence reads, and with no resistance genes detected. No bacterial growth was obtained on CSF culture during hospitalization. The negative CSF culture was likely due to prior antibiotic administration and suboptimal culture conditions. Discontinue intravenous infusion of penicillin G and continue to use ceftriaxone. By day 4 of treatment, his consciousness improved (GCS 15/15), and the endotracheal tube was removed. Enhanced head and neck MRI confirmed a small subdural effusion, C5-C6 spinal cord edema, thickening and enhancement of the anterior and posterior longitudinal ligaments, and meninges at the skull base, consistent with inflammation (Figures 1-2).

Spinal surgery consultation recommended a repeat MRI after two weeks of antibiotics. Endocrinology consultation diagnosed diabetes mellitus, and oral hypoglycemic agents were initiated. Following anti-infective treatment, symptoms improved significantly. To evaluate the therapeutic effect, dynamic reexamination of CSF is required. Repeat CSF analysis showed clear fluid and markedly reduced white cell counts (Table 2).

**Table 2:** CSF test results

Serum test	The first test (2023-11-10）	The second test (2023-11-12）	The third test (2023-11-17）	Reference range	Unit
Color of cerebrospinal fluid	Light yellow	Colorless	Colorless	Colorless	-
Appearance of cerebrospinal fluid	Turbid	Clear and bright	Clear and bright	Clear and bright	-
Cerebrospinal fluid pressure	230	250	138	80-180	mmHg
White blood cells in cerebrospinal fluid	3200	300	30	0	/µL
Red blood cells in cerebrospinal fluid	120	2	80	0	/µL
Protein content in cerebrospinal fluid	1.545	0.576	0.501	0.15-0.45	g/L
Chloride ion content in cerebrospinal fluid	118.4	126.6	123.3	120-130	mmol/L
Glucose content in cerebrospinal fluid	4.49	3.76	3.72	2.5-4.5	mmol/L
Venous blood glucose at the same time	16.1	10.5	7.5	3.9-6.1	mmol/L

Repeat testing on November 20, 2023, revealed that infection markers, liver and kidney function, and electrolytes had basically returned to normal (Table 1). After 10 days of treatment, the patient was discharged to a local hospital for continued antibiotic therapy. A two-week follow-up revealed no active complaints.

## Discussion

*Streptococcus agalactiae* (GBS) is a Gram-positive bacterium commonly colonizing the gastrointestinal tract, respiratory system, and vagina [6]. It is the leading cause of neonatal meningitis and sepsis. Studies suggest the incidence of invasive GBS infection in non-pregnant adults is rising in the USA [7]. Underlying conditions increasing risk include diabetes, cancer, chronic kidney disease, cirrhosis, and immunosuppression [8,9]. Our patient had a history of chemotherapy for neck lymphoma (despite subsequent normal findings, suggesting possible residual immune compromise) and was diagnosed with diabetes during admission, both significant risk factors for invasive GBS disease. Adult GBS infections manifest variably, including bacteremia, meningitis, endocarditis, and splenic abscess [10,11]. Drost et al. reported seven cases of myelitis complicating bacterial meningitis in adults, two of which were GBS-related [12]. Our patient's initial presentation with neck pain followed by trismus and dysphagia raised suspicion for tetanus, prompting specific treatment. However, persistent altered consciousness is atypical for tetanus but characteristic of meningitis. The patient tested positive for meningeal irritation signs, with significantly elevated levels of white blood cells, procalcitonin, and interleukin-6. Infection markers support the diagnosis of bacterial infection. Critically ill patients with elevated procalcitonin may have a poor prognosis. The patient's clinical symptoms, signs, and significantly elevated infection markers all indicate intracranial infection [13,14]. Given the patient's immunocompromised status, there is a possibility of infection with special pathogens that cannot be identified through conventional culture methods. Additionally, some pathogens require prolonged cultivation periods. The patient had previously undergone antimicrobial therapy, which can reduce the positivity rate of pathogen cultures. Therefore, we immediately performed CSF mNGS testing. Guidelines and several studies have reported that timely mNGS testing is necessary for critically ill patients [4,5]. In this case, no pathogens were cultured from the CSF, while mNGS promptly provided a positive result, offering precise guidance for subsequent antimicrobial treatment. It is reported that GBS has a 10% resistance to levofloxacin, tetracycline, and clindamycin; the resistance rate of GBS to penicillin and cephalosporins is 0% [15]. Some studies suggest that β-lactam antibiotics remain the treatment of choice [10]. Ceftriaxone, while used less frequently for GBS than penicillin, offers practical advantages in community settings [16]. In this case, upon receiving the mNGS result, penicillin G was discontinued, and ceftriaxone monotherapy continued, achieving a favorable clinical response.

## Conclusions

The rising incidence of invasive *Streptococcus agalactiae* (GBS) infections in non-pregnant adults, particularly elderly and diabetic populations, warrants increased clinical vigilance. This case demonstrates how GBS meningitis may mimic tetanus after trauma, emphasizing the need for broad differential diagnoses when neurological symptoms evolve atypically. Crucially, mNGS enabled rapid pathogen identification after culture failure (likely due to prior antibiotics), directly guiding targeted therapy. The patient’s significant improvement on ceftriaxone reinforces β-lactams’ efficacy against GBS. Timely diagnosis using advanced diagnostics like mNGS, coupled with appropriate antimicrobials, remains essential for optimizing outcomes in this serious infection.
